# Editorial Note: Genotype-Specific Differences between Mouse CNS Stem Cell Lines Expressing Frontotemporal Dementia Mutant or Wild Type Human Tau

**DOI:** 10.1371/journal.pone.0305843

**Published:** 2024-06-27

**Authors:** The PLOS ONE Editors

After publication of this article [1], concerns were raised about Figure 4. Specifically:

- In Figure 4A, blots are described as having been rearranged from the original, uncropped blots provided in Figure S1 however it was noted that several bands within the published Figure 4A appear different to the corresponding bands in the supporting information (Figure S1):
   ○ In Figure 4A DA9 panel, the wt/wt TG Control CK genotype lane appears similar to the wt/wt TG Control TREwt genotype lane in Figure S1A.
   ○ In Figure 4A CP13 panel, the WT1, P130L1, WT2, P130L3, WT4 and wt/wt CK bands do not appear to correspond to the respective bands provided in Figure S1C.
   ○ In Figure 4A AT8 panel, the wt/wt TG Control CK genotype lanes do not appear to correspond to the respective bands provided in Figure S1C.
   ○ In Figure 4A PHF-1 panel, the left wt/wt TG Control CK genotype lane appears similar to the wt/wt TG Control TREwt genotype lane in Figure S1D.

The *PLOS ONE* Editors contacted the authors regarding these concerns. Regarding the Figure 4A DA9 panel the authors stated the labeling of the bands is correct. The *PLOS ONE* Editors believe an error in the labeling some of the control bands may remain in the published figure, however, wt/wt TG Control CK bands are present in Figure S1A, upholding the conclusions of Figure 4A.

Regarding the CP13 and AT8 panels in Figure 4A, the authors stated the published panels are correct. Underlying blot data with additional lanes to those in Figure S1 were provided to PLOS for review (S1 File). S1 File contains all bands in CP13 and AT8 Figure 4A panels, upholding the conclusions of Figure 4A.

Regarding the PHF-1 panel in Figure 4A, whilst the *PLOS ONE* Editors assessed that the left wt/wt TG Control CK band in the published figure does not appear to correspond to the wt/wt TG Control CK band in Figure S1D, the wt/wt TG Control CK band within Figure S1D upholds the conclusions of Figure 4A.

The *PLOS ONE* Editors issue this Editorial Note to inform readers of the above issues that the journal considers resolved by the underlying data provided in S1 File.

## Supporting information

S1 File Underlying Full Length Blots for Figure 4. (ZIP)
