# Supplementary figures and images for: Editorial Note: Genotype-Specific Differences between Mouse CNS Stem Cell Lines Expressing Frontotemporal Dementia Mutant or Wild Type Human Tau
Source: PLoS One. 2024 Jun 27;19(6):e0305843. doi: 10.1371/journal.pone.0305843 (PMC11210852; doi:10.1371/journal.pone.0305843)

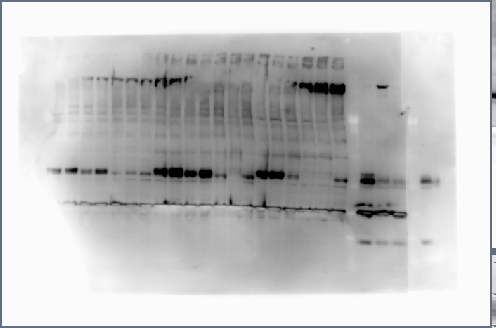

Supplement: S1 File — (ZIP) [file pone.0305843.s001.zip › AT8 for S1 (3).jpg]

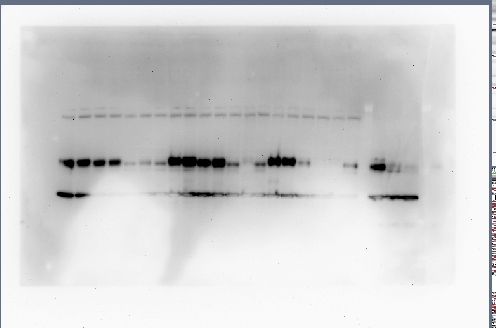

Supplement: S1 File — (ZIP) [file pone.0305843.s001.zip › CP13 for S1 (4).jpg]

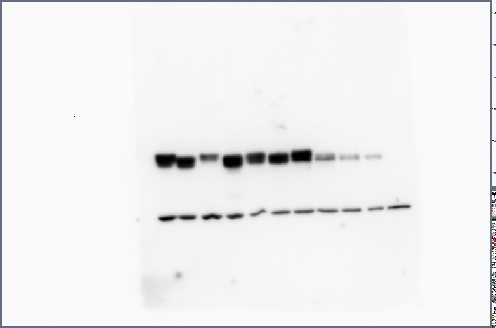

Supplement: S1 File — (ZIP) [file pone.0305843.s001.zip › DA9 for S1 (1).jpg]

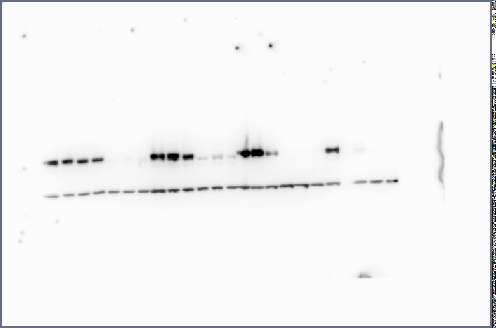

Supplement: S1 File — (ZIP) [file pone.0305843.s001.zip › PHF1 for S1 (3).jpg]

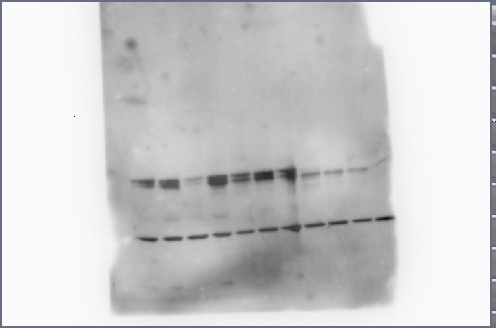

Supplement: S1 File — (ZIP) [file pone.0305843.s001.zip › Tau1 for S1 (1).jpg]

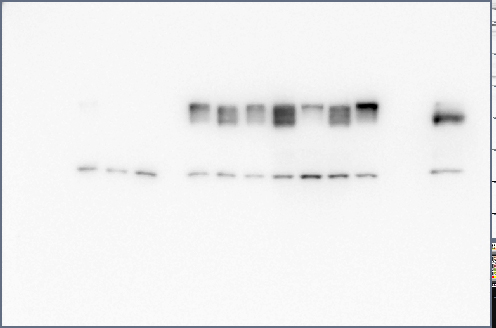

Supplement: S1 File — (ZIP) [file pone.0305843.s001.zip › Tau13 for S1 (1).jpg]
